# Capturing the power of seizures: an empirical mode decomposition analysis of epileptic activity in the mouse hippocampus

**DOI:** 10.3389/fnmol.2023.1121479

**Published:** 2023-05-15

**Authors:** László Molnár, Isabella Ferando, Benjamin Liu, Parsa Mokhtar, József Domokos, Istvan Mody

**Affiliations:** ^1^Department of Electrical Engineering, Sapientia Hungarian University of Transylvania, Târgu-Mures, Romania; ^2^Department of Neurology, The David Geffen School of Medicine at UCLA, Los Angeles, CA, United States; ^3^Department of Neurology, School of Medicine at University of Florida, Miami, FL, United States

**Keywords:** epileptogenesis, electrographic seizure, inter-ictal spike, dentate gyrus, EMD analysis, pHFOs

## Abstract

**Introduction:**

Various methods have been used to determine the frequency components of seizures in scalp electroencephalography (EEG) and in intracortical recordings. Most of these methods rely on subjective or trial-and-error criteria for choosing the appropriate bandwidth for filtering the EEG or local field potential (LFP) signals to establish the frequency components that contribute most to the initiation and maintenance of seizure activity. The empirical mode decomposition (EMD) with the Hilbert-Huang transform is an unbiased method to decompose a time and frequency variant signal into its component non-stationary frequencies. The resulting components, i.e., the intrinsic mode functions (IMFs) objectively reflect the various non-stationary frequencies making up the original signal.

**Materials and methods:**

We employed the EMD method to analyze the frequency components and relative power of spontaneous electrographic seizures recorded in the dentate gyri of mice during the epileptogenic period. Epilepsy was induced in mice following status epilepticus induced by suprahippocampal injection of kainic acid. The seizures were recorded as local field potentials (LFP) with electrodes implanted in the dentate gyrus. We analyzed recording segments that included a seizure (mean duration 28 s) and an equivalent time period both before and after the seizure. Each segment was divided into non-overlapping 1 s long epochs which were then analyzed to obtain their IMFs (usually 8–10), the center frequencies of the respective IMF and their spectral root-mean-squared (RMS) power.

**Results:**

Our analysis yielded unbiased identification of the spectral components of seizures, and the relative power of these components during this pathological brain activity. During seizures, the power of the mid frequency components increased while the center frequency of the first IMF (with the highest frequency) dramatically decreased, providing mechanistic insights into how local seizures are generated.

**Discussion:**

We expect this type of analysis to provide further insights into the mechanisms of seizure generation and potentially better seizure detection.

## Introduction

Seizures are the hallmark of epilepsies that affect over 50 million people worldwide (Guekht et al., 2021). Temporal lobe epilepsy (TLE), is the most common form of pharmaco-resistant epilepsy and is associated with significant morbidity and mortality due to recurrent and unpredictable seizure onset (Nayak and Bandyopadhyay, 2022). There is significant correlation between the occurrence of a neurological insult earlier in life (head trauma, status epilepticus, stroke, inflammation, etc.) and the development of TLE after a “latent period” (Maguire, 2016). Little is known about the process that leads to the development of the chronic epileptic state. It has been hypothesized that a likely biomarker of this transitionary (epileptogenic) period is the occurrence of high frequency neuronal oscillations not generally encountered in the dentate gyrus, termed pathological high-frequency oscillations (pHFO) (Engel et al., 2009, 2013). Neuronal networks in the normal hippocampus and parahippocampal structures of humans and animals generate high-frequency oscillations (HFOs) in the 80–200 Hz range (Engel et al., 2009; Jacobs et al., 2012; Jefferys et al., 2012). Such local field potential (LFP) oscillations are believed to facilitate information transfer by synchronizing neuronal activity over long distances. Higher frequency oscillations in the range of 200–600 Hz are generally pathological and are readily recorded from the hippocampus and parahippocampal structures of patients with TLE, as well as in rodent models of epilepsy even during epileptogenesis (Bragin et al., 1999, 2002; White et al., 2006; Engel et al., 2009; Jacobs et al., 2012; Jefferys et al., 2012). Hippocampal pHFO exhibit a dynamic evolution during the epileptogenic period following status epilepticus (SE), consistent with the hypothesis of their role in the transition from a healthy state to the chronic state of TLE (Jones et al., 2015). In addition to the pHFO, the epileptogenic hippocampus also exhibits short focal seizures that typically cannot be recorded with surface electrodes. There is little known about the frequency components of these early purely electrographic seizures which may serve as a powerful biomarker for early detection of seizure onset. In this study we examine the individual frequency components of these early focal events during epileptogenesis using the empirical mode decomposition (EMD) method (Huang et al., 1998).

## Materials and methods

These experiments were carried out in accordance to ARRIVE guidelines.

### Mice

In this study we used adult (15–20 weeks-old) female (*n* = 4) and male (*n* = 8) C57BL/6 J mice. Mice were housed with *ad libitum* access to food and water and kept on a 12-h light/dark cycle (from 6 AM to 6 PM), under the care of the UCLA Division of Laboratory Animal Medicine (DLAM) or in the recording cages during continuous recordings. All recordings were performed continuously for at least 3 days according to a protocol approved by the UCLA Chancellor’s Animal Research Committee.

### Surgeries

Surgeries were performed under aseptic conditions on mice weighing 25–30 g under isoflurane anesthesia (2–2.5% in 100% O_2_) as previously described (Barth et al., 2014; Vrontou et al., 2022). Body temperature was maintained at 37°C using a rectal probe and a water-circulated heating pad. Electrodes were implanted bilaterally into the hilus of the dentate gyrus (−2.3 AP; 1.6 ML; 2 DV). Next, 70–75 nL of 20 mM KA (Tocris, in 0.9% sterile saline) was stereotaxically injected just above (Bedner et al., 2015; Jones et al., 2015) either the right or left dorsal hippocampus using the following coordinates (from Bregma, in mm): −1.9 AP, −1.5 ML and 0.8 DV. Status epilepticus (SE) typically began 30 min to 1 h post-surgery, after recovery from anesthesia. Two hours following the onset of SE, mice were injected with lorazepam (6 mg/kg) to abort the seizures.

### Chronic recordings

Four to 5 days after the animals had fully recovered from the surgery, chronic simultaneous video and local field potential recordings were carried out continuously (24 h a day) for 1–8 weeks as previously described (Molnár et al., 2018; Vrontou et al., 2022). LFPs were recorded with a custom-made miniature dual headstage amplifier connected to the electrode sockets mounted on the animal’s head and then wired to an electrical commutator (Dragonfly Inc.). The signals from the commutator were fed through a 16-channel amplifier (A-M Systems model 3,500) with a gain of 1,000. Signals were low-pass filtered at 600 Hz and sampled at 2048 s^−1^ per channel, using a 16 bit National Instruments A/D converter board. Continuous data acquisition was carried out using Igor NIDAQ tool (Wavemetrics, Lake Oswego, OR, United States). No electrographic seizures were recorded before day 7 after the KA administration. Our analysis included only electrographic seizures (from seizure numbers 1–5 within a given animal) without generalization to the tonic–clonic phase.

### Data analysis

All data analyses were carried out using custom written procedures in IgorPro technical graphing and data analysis software, version 6.37–9 (Wavemetrics, Lake Oswego, OR, United States) using its built-in functions for RMS measurement, FIR filtering, FFT, Hilbert amplitude, continuous wavelet transform, etc. Further details about data analyses can be found in the following sections.

### Seizure and post-ictal lull detection

To detect seizures, we started with using the Autocorrelation method presented in White et al. (2006). The continuous 12 h epochs of our data records were sampled at 2048 s^−1^. For seizure detection we down-sampled the signal to 256 s^−1^. We defined basic time epochs of 125 ms (instead of 120 ms used by White et al., 2006), each one containing 32 points from the down-sampled signal. In each 125 ms interval, we calculated the maximum and the minimum of the signal, obtaining the *Max_wave* and *Min_wave* functions. Using these values, we calculated *HV* and *LV* as follows:


$$HV\left[i\right]=\min\left(\max\_\text{wave}\left[i\right],\max\left(\begin{array}{l} \max\_\text{wave}\left[i+1\right],\\ \max\_\text{wave}\left[i+2\right] \end{array}\right)\right)$$

and


$$LV\left[i\right]=\max\left(\min\_\text{wave}\left[i\right],\min\left(\begin{array}{l} \min\_\text{wave}\left[i+1\right],\\ \min\_\text{wave}\left[i+2\right] \end{array}\right)\right)$$

In this way, we considered the correlation of the successive time epochs, avoiding the processing of any spurious noise. Next, we defined a 5 s long window sliding in steps of 1 s in which we summarized the HV-LV difference (40 points in every 5 s). We calculated *Metric3S* as follows:


Metric3S=sum(HV−LV)

We used the *Metric3S* values to define epochs to be considered seizures (and post-ictal lulls) in the record. Previously we described an original approach to establish objective thresholds using only the intrinsic properties of the extracted signals (Molnár et al., 2018). We enhanced the autocorrelation method using this thresholding technique. The all-point histograms of the *Metric3S* values were comprised of two normal distributions that were fitted separately. The bimodality of the overall distribution was tested using the formula below (Ashman et al., 1994) to verify that the value *D* > 2, where


$$D\equiv \frac{\left|{\text{mean}}_{1}-{\text{mean}}_{2}\right|}{\sqrt{\frac{SD_{1}^{2}+SD_{2}^{2}}{2}}}>2$$

Once the bimodality of the two distributions was verified, we set the threshold for seizure detection as the *Metric3S* values larger than the mean of the leftmost Gaussian + 3*SD. Epochs where the value of *Metric3S* exceeded this objectively determined threshold were considered to be part of a seizure. Detec ted seizures with a duration of <5 s were discarded. Seizures are usually followed by periods with very low amplitude oscillations. We named these segments post-seizure lulls, and defined them by the *Metric3S* values lower than the mean of the leftmost Gaussian – 3*SD.

### Inter-ictal spike detection

For IIS detection we used the original recordings sampled at 2048 s^−1^. We defined a 30 ms window sliding in steps of 25 ms. In each of these segments we calculated the difference between the maximum and minimum values, obtaining a difference signal. The all-point histogram of this difference signal also had a bimodal distribution with two Gaussian curves best fitting the distribution. After checking for the distributions satisfying bimodality, as above, values larger than the mean of the right-most Gaussian + SD were considered as the threshold for IIS detection. We ignored IIS that were detected during detected seizures.

### Empirical mode decomposition

The application of decomposition methods to time series is an important analysis step allowing patterns and behaviors to be extracted as components providing insight into the oscillatory mechanisms underlying the time series. The most important is that the decomposition methods provide components which are physically meaningful. The Empirical Mode Decomposition (EMD) introduced by Huang et al. (1998) is a powerful method highly suitable for analyzing nonlinear and non-stationary data, as intracortical LFP, in an adaptive manner. The key part of the EMD analysis is the extraction from any complicated time-series data of a finite set and often small number of intrinsic mode functions (IMFs) that can produce well-behaved Hilbert transforms. This decomposition method is adaptive and since the decomposition is based on the local characteristic time scale of the data, it is applicable to nonlinear and non-stationary processes.

- An intrinsic mode function (IMF) is a function that satisfies two conditions (Huang et al., 1998):
- in the whole data set, the number of extrema and the number of zero crossings must either be equal or differ at most by 1;
- at any point, the mean value of the envelope defined by the local maxima and the envelope defined by the local minima is 0.
- The output of the EMD algorithm applied for a time series x(t) is a set of n IMFs (${\text{imf}}_{i}\left(t\right),i=\bar{1,n}$) and a final residual, r(t), where the sum of the individual IMFs and the final residual gives the original time series (Kim and Oh, 2009; Paul, 2018):
$$x\left(t\right)=\sum_{i=1}^{n}{\text{imf}}_{i}\left(t\right)+r\left(t\right)$$

There are two different stopping criteria for the EMD algorithm: the first one is based on definition of an IMF, and the second based on the number of IMFs to be extracted. The extracted IMFs preserve the inherent properties of the original signal. As the decomposition level increases, the complexity of the IMFs decreases, and so does the scale of the signal. When there are abnormal activities in brain signals, the IMFs will show different behavior than during normal brain activities. Therefore, various features can be extracted from the IMFs and even the IMFs themselves can be used as features for seizure detection from EEG signals (Paul, 2018). However as presented in Bonizzi et al. (2014) IMF components retrieved by EMD may be affected by mode mixing.

The Hilbert spectral analysis is used to determine the instantaneous frequency (IF) as function of time for the extracted IMFs. The IF is the frequency of the signal determined at an instant of time t. In classical Fourier analysis, a complete oscillation of a sine or cosine function is needed to find out the local frequency, but it makes little sense for non-stationary signals like the EEG. The obtained frequency–time distribution of signal amplitude or energy allows the identification of highly localized features in the signal. We have not used this analysis here, but for short segments of the raw signals we resorted to using the Morlet wavelet analysis as previously described (Jones et al., 2015). The Morlet mother wavelet was used, and scales were chosen to reflect frequencies between 100 and 700 Hz in steps of 1 Hz using ω_0_ = 6. Z-scored wavelet transforms were calculated based on the entire time-frequency matrix to represent relative changes in magnitude. Warmer colors represent greater Z-scores on a scale of 0–10.

We performed the EMD analysis according to a published approaches (Huang et al., 1998; Wang et al., 2010). Initially, to extract the IMFs, 7 s long recording epochs were used. The number of iterations (sifting steps) to obtain the IMFs was stopped when a stopping criterion was reached. For stopping the sifting steps we used the S-number criterion. The S-number is defined as the number of consecutive sifting iterations in which the number of zero-crossings and extrema are the same and differ at most by one. We used S = 2.

We calculated the spectral properties (magnitude squared) of each IMF, using the Fast Fourier Transform (FFT) for each IMF obtained by the EMD analysis. We defined spectral boundaries for each IMF spectrum, defining a rectangle with the area equal to the area of the spectrum resulting from FFT analysis (see below). In this way, a maximal and a minimal value of the frequency spectrum could be identified. The center of the rectangle was used to define the central frequency of the IMF spectrum. The spectral power of the IMFs we calculated by obtaining RMS of the magnitude squared of the entire FFT spectrum for a given IMF.

**Figure 1:**
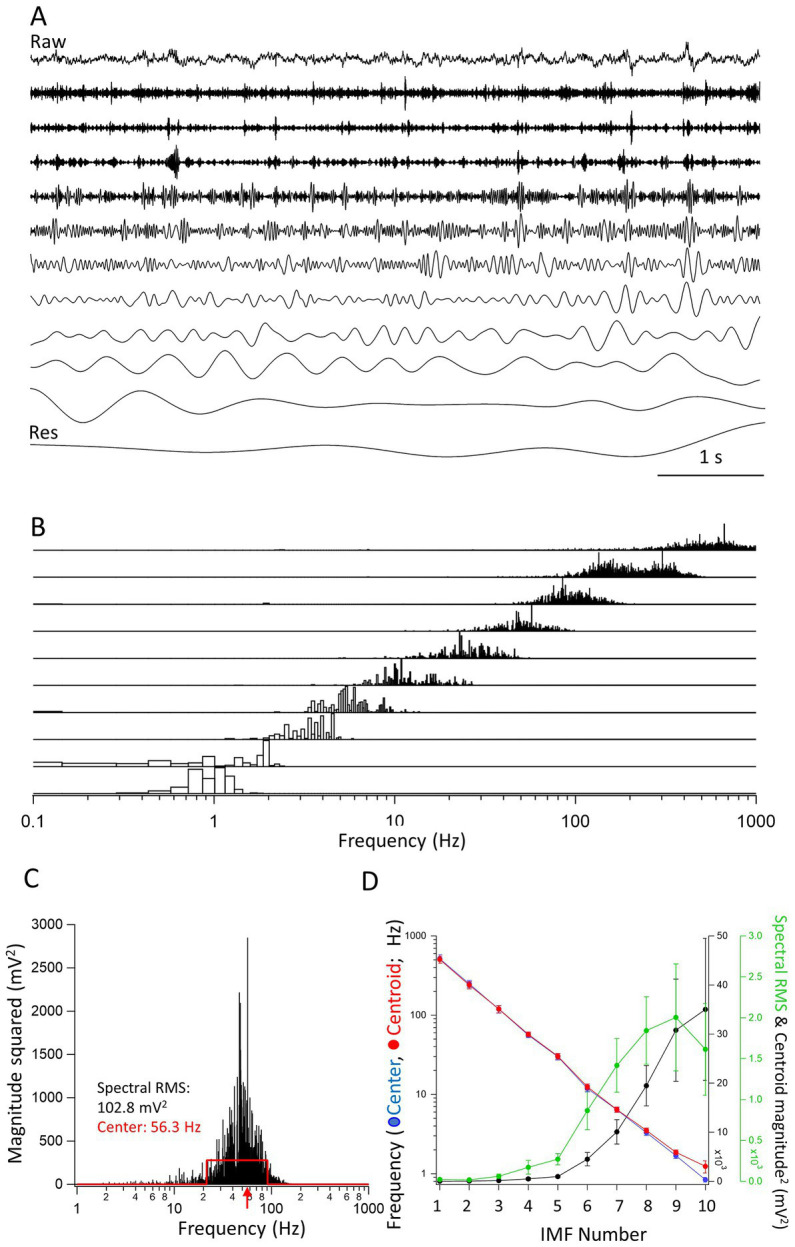
Properties of the intrinsic mode functions (IMFs). **(A)** A raw trace (top) of a 7 s long segment from an inter-ictal period. The IMFs (1–10) are shown under the raw trace. The last trace is of the residual (Res), left after all IMFs have been obtained. Vertical scale bars are not included in the figure as all traces are shown relative to their largest peak-to-peak deflections. **(B)** The frequency spectra of the IMFs (1–10) obtained through FFT of the individual IMFs. Note the overlap in the frequency spectra. The spectra were expressed as magnitude squared and were normalized for each IMF to the largest value of the spectrum. **(C)** The spectrum of IMF4 is used to illustrate the calculation of the center frequency. The red traces define a rectangle with an equal area to the spectrum and with a maximal overlap in area with it. The center frequency (the middle of the rectangle) is shown by a red arrow at 56.3 Hz. The left and right sides of the rectangle were used to define the minimum and the maximum frequencies of the IMF, respectively. The RMS value of the FFT spectrum, which we took as the spectral power of the IMF, is also indicated (102.8 mV^2^). **(D)** The center frequencies as calculated by the rectangle method (*red* •), the frequencies of the centroids (*blue* •), the spectral RMS (*green* •), and magnitude squared value of the centroid (*black* •) are plotted as averages from 7 s long epochs determined in separate recordings from 8 mice. There is a clear linear decrease on the log scale of the frequencies indicating an exponential decline in the center and centroid frequencies of the successive IMFs. Note that the largest powers are carried by frequencies between approximately 1–10 Hz.

To improve the temporal accuracy for the localization of the onset and termination of the seizures, we reduced the length of analysis intervals, observing, that EMD analysis can be performed, and gives very accurate results even in 1 s long intervals. Longer intervals (e.g., 7 s) were used to analyze recordings during inter-ictal periods (e.g., Figure 1) to obtain the center and centroid frequencies of the IMFs, their spectral RMS and the power of the IMF waveforms. The rest of the EMD analysis was standardized by using 1 s long intervals, during equal duration pre-seizure, seizure, and post seizure epochs.

## Results

### Empirical mode decomposition analysis reveals exponentially decreasing frequency components of the IMFs derived from The LFP signals

A given recording segment usually yielded 8–10 separate IMFs and a residual component (Figure 1A). The IMFs obtained through EMD analysis have frequency spectra that overlap from one IMF to the next. As shown in the normalized spectra in Figure 1B, the frequency spectra of successive IMFs was obtained by performing a FFT on each IMF separately. It is clear from the figure that there are overlapping frequencies of the spectra that depend on the unbiased extraction of the IMFs. As comparing the parameters such as peaks or areas of the FFT spectra are somewhat less informative, we devised an objective method to find the center frequencies and high- and low-end values of the spectra. As illustrated in Figure 1C, the method consists of finding a rectangle with a similar area as the FFT spectrum of the given IMF. The sides of the rectangle are automatically adjusted by the algorithm until a maximum overlap in area is found between the FFT spectrum and the rectangle. Then, the frequency given by the x-value, i.e., the frequency at the center of the rectangle is the center frequency of the IMF, and the positions of the left and right edges of the rectangle constitute the minimum and maximum frequencies, respectively of the given IMF. We also calculated the centroid of the FFT distribution. The coordinates of the center of gravity were calculated using the integral method. The value of the abscissa constituted the centroid frequency while the value of the ordinate was considered the magnitude squared of the centroid. The spectral RMS was calculated as the RMS of the FFT of the spectrum. Once all these values for all the IMFs through EMD analysis were obtained, we could plot the average values obtained from n = 8 mice of the successive IMFs in recording segments of 7 s during inter-ictal periods. As shown in the Figure 1D, when plotted on a log-scale ordinate, the decrease in the center and centroid frequencies of the IMFs is nearly perfectly exponential, i.e., a straight line on the logarithmic scale. This resembles very much the logarithmic relationship between the frequencies of named brain rhythms (Buzsáki and Draguhn, 2004). On the same figure we also plotted the spectral RMS values and the magnitude squared of the FFT centroid for each of the IMFs. As expected, the largest spectral powers are found in IMFs with their center frequencies of 1–20 Hz.

### Empirical mode decomposition analysis reveals diminished frequency components of and enhanced power of the IMFs during seizures

We next wanted to know how the center frequencies and the spectral power (RMS) of the IMFs are affected during electrographic seizures when compared to the pre-seizure (interictal) periods. We recorded 15 electrographic seizures in 4 mice starting with the first recorded seizure that met our seizure definition criterion (please see Materials and Methods). In all animals (n = 8), the first seizures were always recorded during the 12 h of the dark cycle (6 PM to 6 AM) 6–8 days after the suprahippocampal injection of kainic acid. In one animal we also recorded 1 and 2 seizures, respectively, during the following 12 h of light cycle and the dark cycle after that. We wanted to concentrate on the earliest seizures during the period of epileptogenesis. The duration of the seizures (n = 20 in n = 8 animals) was variable (range: 10–72 s) with an average (± SD) duration of 28.3 ± 18.1 s. For the EMD analysis we used a recording segment that contained the seizure, as detected from beginning to end, and a pre-seizure period of equal duration, and a post seizure period of equal duration. Thus, the entire recording used for the EMD analysis was three-times the duration of the detected seizure. The EMD analysis was then carried out on non-overlapping 1 s long segments. Therefore, the time resolution of the center frequencies and the spectral RMS values for each IMF was 1 s. This approach provided sufficient resolution to examine in detail the time course of the changes in these two parameters before, during and after the seizures.

The variability in the duration of seizures and consequently the recorded epochs used for analyses precluded us from simply time-averaging across all recordings. We resorted to calculating the relative times based on the total duration of a given seizure. We opted to divide the total length of the seizure into 5 parts (quintiles). This division automatically generated quintiles also for the pre-seizure and the post-seizure periods. The center frequencies and spectral RMS values during the quintiles were averaged from the 1 s epochs that fell within the quintiles. In this manner, for each recording containing the seizure and an equal duration of pre- and post-seizure period we obtained 15 equidistant points (in relative seizure quintile time) covering all three phases of interest during the recordings. Once these calculations were done for all the recorded seizures, the quintiled data could be averaged across all recordings and the center frequencies and RMS values could be compared during the pre-seizure, seizure, and post-seizure periods.

**Figure 2:**
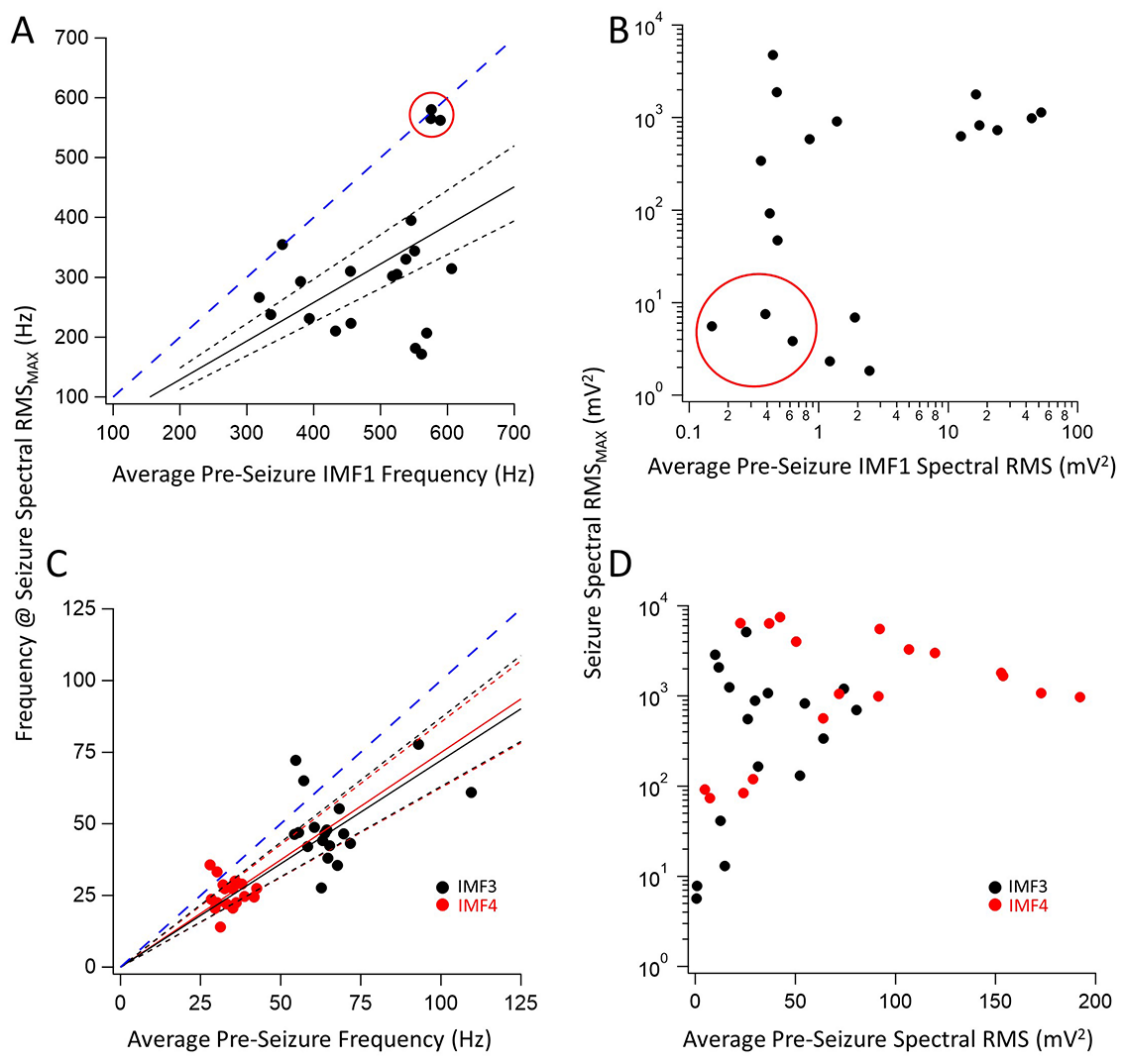
Relationship between pre-seizure and during seizure center frequencies and spectral RMS of three key IMFs. **(A)** Plot of the IMF1 center frequency during the maximum RMS found in a quintile of a seizure for 20 seizures vs. the corresponding pre-seizure average center frequency of IMF1. The three data points circled in red were identified as belonging to a separate cluster by the farthest point clustering algorithm (see text for details). A linear regression line through the origin (*black solid line*) was fitted to the remaining 17 points resulting in a fitted slope (± SD) of 0.64 ± 0.04 indicating a decrease in the IMF1 frequencies during seizures. The 95% confidence intervals for the linear fit are shown with dotted lines and show that the slope of the fit is significantly different than a slope of 1.0 (*blued dashed line*) that would indicate no change in the frequencies during seizures when compared to the pre-seizure period. **(B)** Plot of the largest spectral RMS of IMF1 during a seizure time quintile vs. the pre-seizure average spectral RMS values. Note that the three events circled in **(A)** have the smallest spectral RMS values both during the seizures and the pre-seizure periods. As the center frequencies of IMF1decreased during seizures, the spectral RMS increased significantly, sometimes by an order of magnitude or more. **(C)** Same plots as in **(A)** but for IMF3 (*black symbols*) and IMF4 (*red symbols*). The slopes (± SD) of the fitted lines are given in the text. Both slopes were significantly different that a slope of 1.0 (*blue dashed line*) as indicated by the 95% confidence intervals (*dotted lines*). **(D)** Same plots as in **(B)** but for IMF3 (*black symbols*) and IMF4 (*red symbols*). These two IMFs represent those with the largest spectral RMS increases during seizures, sometimes reaching over a 100-fold changes.

We were particularly interested in the IMFs that showed the largest changes in center frequencies and RMS values during seizures compared to the pre-seizure periods. For each IMF, we first identified the quintiles with the largest values of the power (RMS) during the seizure segment. We chose the center frequency of the same IMF during this same quintile to be compared to the average center frequencies of the same IMF during the pre-seizure period. Figure 2 shows the center frequencies (Figure 2A) and spectral RMS values (Figure 2B) for IMF1 (the IMF with the largest center frequency) plotted as the center frequency of the quintile with the largest spectral power during the seizure vs. the average center frequency during the pre-seizure period for all recorded seizures. A clustering analysis using farthest-point clustering algorithm set to identify two clusters classified the three values circled with red in the figure as an independent cluster. Interestingly, these values were all obtained from the first 3 seizures of animal I0001 and were excluded from the linear fitting of the data. A straight line through the origin fitted to the 12 remaining seizures indicates that the frequencies of IMF1s were reduced by average to 64% (the slope of the linear fit ± SD is: 0.64 ± 0.04) of the values compared to the center frequencies of the pre-seizure periods. The 95% confidence intervals (black dotted lines) show that the slope of this line is significantly different from the line with a slope of 1 (blue dotted line). Considering all 20 seizures, the pre-seizure average (± SD) IMF1 frequency was 491.5 ± 92.7 Hz while the average frequency at maximum RMS during the seizure was 319.3 ± 123.3 Hz. This difference is significantly different (two tailed *p* = 0.00003) as measured by a nonparametric Wilcoxon paired rank test. Similarly, as shown on Figure 2B, the pre-seizure spectral RMS average of IMF1s was 9.4 ± 11.6 mV^2^ vs. the average of the maximum spectral RMS values during the seizures of 774.5 ± 812.4 mV^2^, also significantly different (two tailed *p* = 0.0001) according to the nonparametric Wilcoxon paired rank test.

We have also identified the two IMFs that had the largest fractional increase in power during the seizures. These were IMF3 and IMF4. The changes in their frequencies for the 20 seizure and pre-seizure periods are shown in Figure 2C (IMF3: black symbols, IMF4: red symbols). As done in Figure 2A, we fitted linear regression lines to the data points to obtain the slopes of the relationship between the frequencies of the IMFs during the pre-seizure period vs. those at the time quantiles during seizures with the highest spectral RMS values. These slopes (± SD) for the fitted lines were, for IMF3: 0.72 ± 0.09 and nearly identical for IMF4: 0.75 ± 0.08. The dotted 95% confidence interval lines indicate that the slopes are significantly less than the slope of unity (dotted blue line). For all seizures recorded, the pre-seizure average (± SD) IMF3 frequency was 66.9 ± 13.8 Hz while the average frequency at maximum spectral RMS during the seizure was 42.3 ± 12.7 Hz. The same values for IMF4 were 34.2 ± 4.2 Hz and 25.9 ± 5.1 Hz, respectively. This decrease in frequency during the seizure is significantly different for both IMFs (two tailed *p* = 0.0031 for IMF3 and *p* = 0.0013 for IMF4) as derived from nonparametric Wilcoxon paired rank tests. The increase in the spectral RMS of the two IMFs during the seizures was remarkable. Figure 2D depicts the large increases in the power of IMF3 and IMF4 during seizures compared to the pre-seizure periods. For some seizures these increases were 2–3 orders of magnitude. The pre-seizure spectral RMS average of the IMF3s was 31.1 ± 24.4 mV^2^ vs. the average of the maximum spectral RMS values during the seizures of 1883.5 ± 2901.9 mV^2^, whereas for the IMF4s these values were 79.7 ± 58.9 mV^2^ and 2476.1 ± 2478.7 mV^2^, respectively. The large increases in the spectral power of IMF3 and IMF4 during seizures compared to the pre-seizure averages were both significantly different (two tailed *p* = 0.0001 for IMF3 and *p* = 0.00007 for IMF4) according to nonparametric Wilcoxon paired rank tests.

### IMF1 frequency decrease during seizures and the presence of pathological high-frequency oscillations

**Figure 3:**
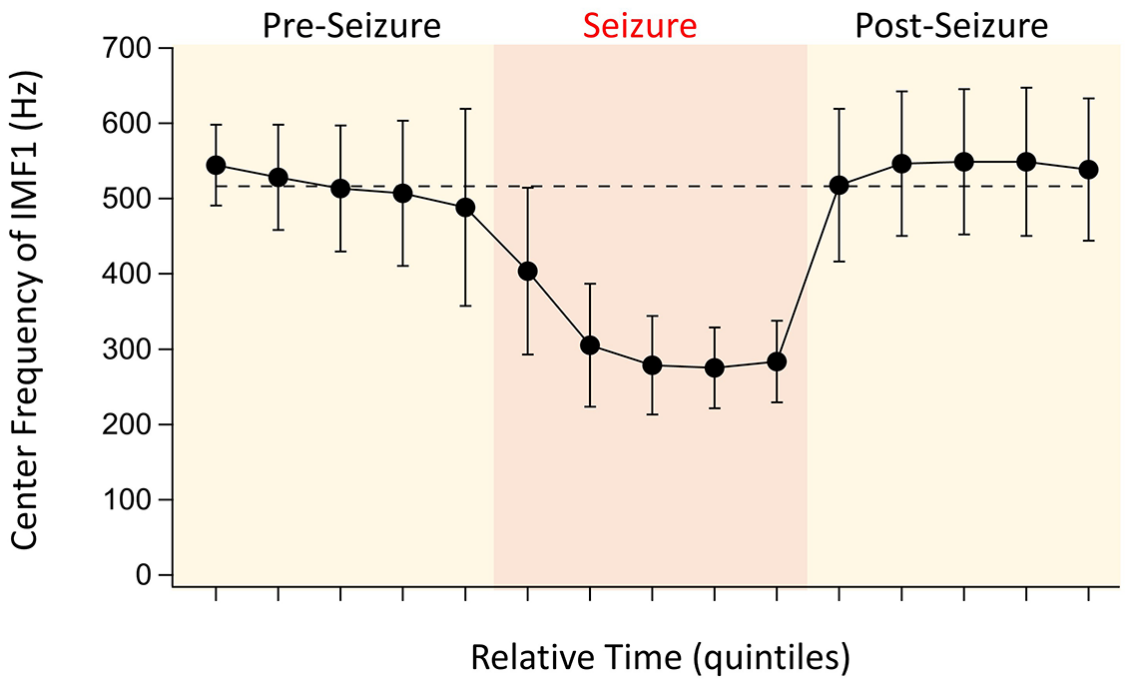
Evolution of IMF1 center frequencies during the pre-seizure, seizure, and post seizure periods. To have a relative comparison of the time courses for the various seizures recorded in different animals, the recordings were divided into five quintiles of time for each of the three periods that were of similar duration (see text for details). The points are the absolute values of the average center frequencies of IMF1s that were chosen based on their average center frequencies ≥400 Hz during the pre-seizure periods (*n* = 17 seizures, 8 animals). The error bars represent SD. Note the large decrease in center frequencies during the seizures, already starting at the end of the pre-seizure period, and the quick recovery of the center frequencies once the seizures are over.

The first IMF derived through EMD, IMF1 is the component of the signal that has, by definition, the highest frequency. As the built-in filter of our 16-channel amplifier had a broad roll-off, our signals low-pass filtered at 600 Hz still had considerable power at this frequency. As we have shown above (Figure 2A), in 17 of 20 detected seizures, the frequencies of the IMF1s were substantially reduced during seizures when compared to the pre-seizure periods. After the seizures, these frequencies quickly returned to the pre-seizure values even when the peak-to-peak amplitudes of the LFPs were reduced during the post-seizure lull periods. The average values for the changes of the IMF1 frequencies for the 17 remaining seizures during the time quintiles determined from the seizure durations are shown in Figure 3. As shown in the figure, it is remarkable how rapidly the IMF1 frequency declines at the beginning of the seizure, perhaps even before this time. Conversely, after the seizure ends, the IMF1 frequency returns to pre-seizure levels.

**Figure 4:**
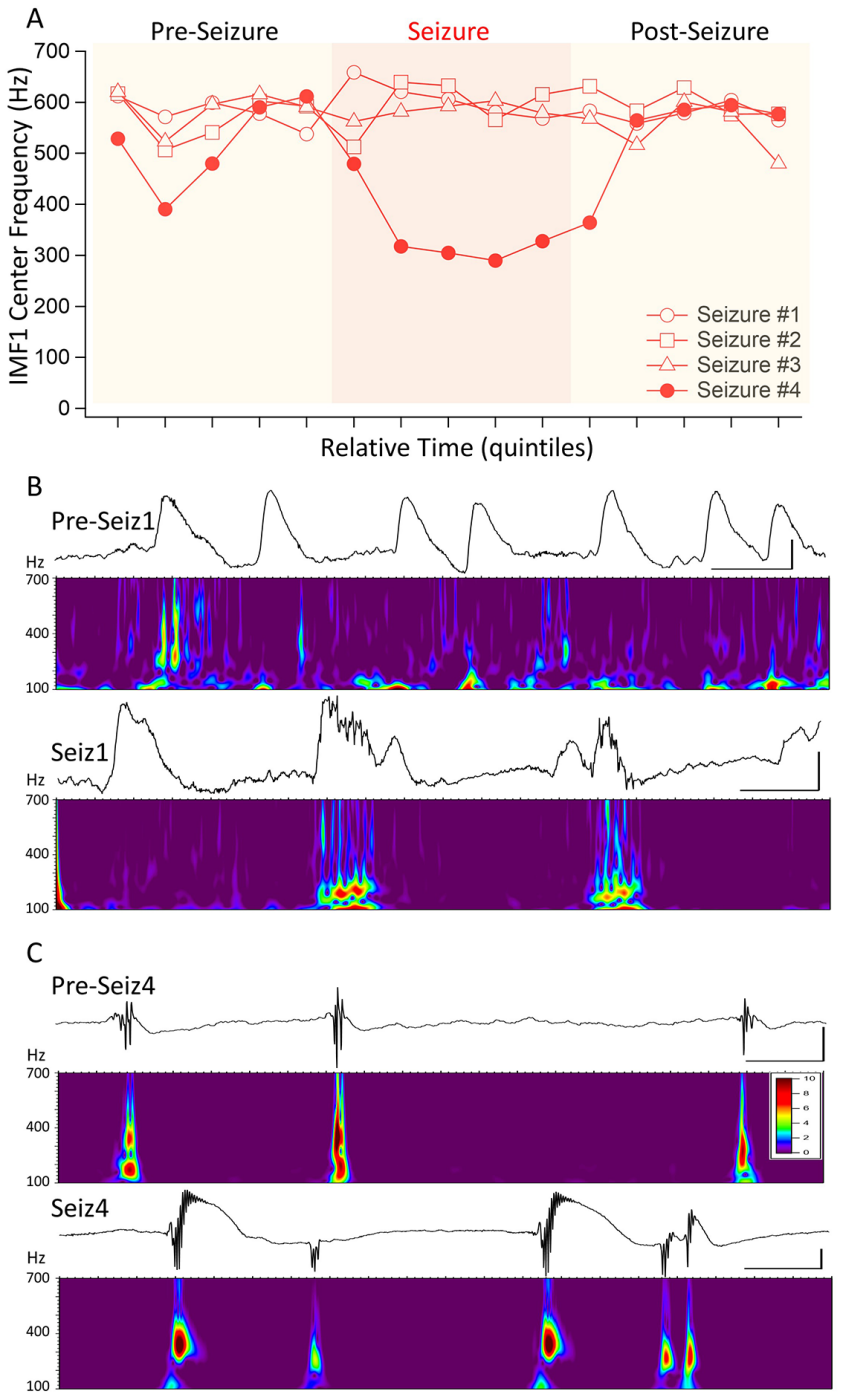
Distinguishing four seizures in the same animal based on the changes of IMF1 center frequencies during the seizures. **(A)** A plot similar to that in Figure 3, except for four consecutive seizures in mouse #I0001. The first three of these seizures were identified as a cluster apart from the rest (red circle in Figure 2A) and show no decrease in center frequencies during the seizures. The fourth (Seizure #4) shows the typical decrease in center frequency during the seizure. **(B)** Inter-ictal spikes (Pre-Seiz1) and ictal spiking (Seiz1) preceding and during Seizure #1. Below the raw traces the Morlet wavelet time-frequency analyses performed between 100 and 700 Hz (steps of 1 Hz) show the lack of pHFO in these recordings both before and during the seizures. The high-frequency oscillations present pre-ictally are also there in periods between the spikes and are most likely do to muscle contractions. The frequencies with the highest amplitudes found during ictal spiking have frequencies of <200 Hz. **(C)** The same as in **(B)**, but for Seizure #4, which showed a significant decrease in center frequencies during the seizure. As shown by the Morlet wavelet analysis below the raw traces, both pre-ictal spikes, i.e., IIS (pre-Seiz4) and the ictal spiking (Seiz4) have very strong components in the 400 Hz range corresponding to pHFO. The color scale indicates the Z-score of the Morlet matrix and is the same scale for **(B,C)**.

The 3 points in Figure 2A that were identified by the cluster analysis to belong to a different cluster of data were those seizures in which the pre-seizure central frequencies of IMF1s did not decrease. As we had a fourth seizure from the same animal in which the IMF1 frequency readily decreased during the seizure period, we wanted to conduct a more detailed analysis of the 4 seizures recorded in this mouse (I0001). The center frequencies of the IMF1s during the time quantiles of the four seizures are plotted in Figure 4A. The numbering of the seizures reflects their order of occurrence in the animal. Seizure #1 was the first electrographic seizure recorded in this mouse during a dark cycle period, and the next 2 seizures (Seizures #2–3) were also recorded during the same 12 h. Seizure #4 was the next seizure, and it was recorded during the light cycle of the following 12 h. As show in the figure, there is a remarkable difference between Seizures #1–3 and Seizure #4 regarding the central frequency of IMF1 during the seizure. To investigate potential differences in the two types of seizure (with decreased central frequency during seizure and without) we analyzed the raw signals between 100–700 Hz using Morlet wavelets to obtain a high-resolution image of the time-dependent changes in signal frequency. As illustrated in the example of Seizure #1 in Figure 4B, we checked for high frequency components in the signals during inter-ictal spikes (IIS) present during the pre-seizure period and during large spikes recorded during the seizures themselves. In all these events, the highest frequencies of the LFP recordings did not surpass ~200 Hz. The occasional higher frequencies were also present between IIS and most likely correspond to muscle contraction artifacts. This limited frequency spectrum of the IIS and the ictal spiking was also there in the Seizure #2 and #3 recordings. In sharp contrast, when we examined the pre-seizure IIS and the ictal spikes of Seizure #4, the seizure with the substantial reduction in IMF1 center frequency during the seizure, we found very clear high frequency components of the LFP signals during these events (Figure 4C). The frequencies ranged between 350–450 Hz, much like those of the pathological high-frequency oscillations (pHFO) characteristically recorded in epileptic foci both in human epileptic patients and in animal models of the disease (Bragin et al., 2002; Engel et al., 2009; Ogren et al., 2009; Jacobs et al., 2012; Jefferys et al., 2012).

**Figure 5:**
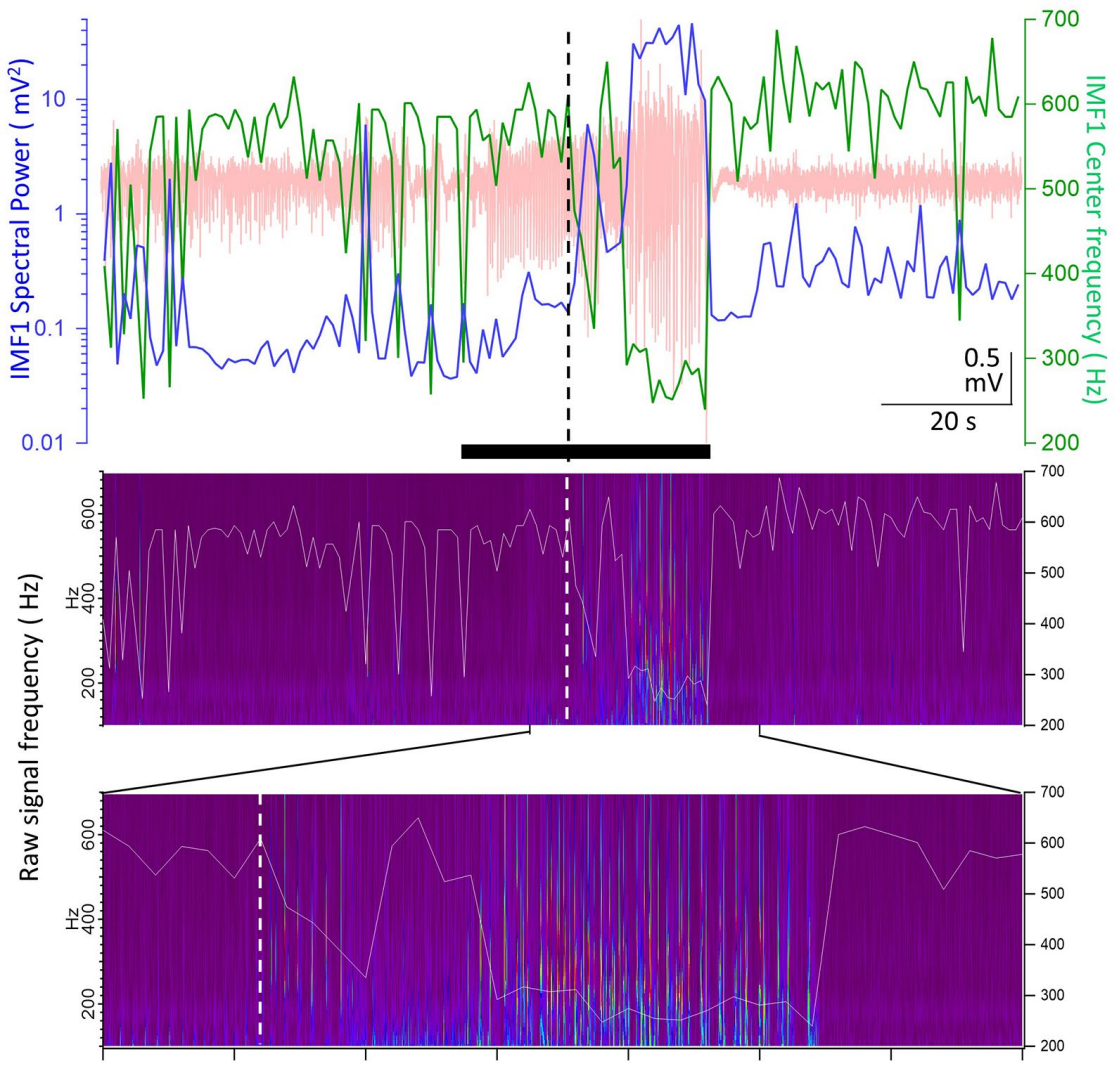
Example of a seizure recorded in animal #I0012 where the IMF1 frequency starts to decrease after seizure onset. Top panel: plot of the IMF1 spectral power (*blue line, left y-axis*) and center frequencies (*green line, right y-axis*) at 1 s resolution during the entire pre-seizure, seizure, and post-seizure periods (the raw record is indicated in *pale red*). The black bar below the trace shows the seizure duration as detected. The vertical dashed line shows the point where the center frequency of IMF1 starts to decrease during the seizure. Middle panel: the Morlet wavelet transform of the raw recording indicating frequencies between 100 and 700 Hz (*left y-axis*). Superimposed on the image is the trace of the center frequency of IMF1 (*right y-axis*). Bottom panel: expanded region as indicated by the marks, showing the time during the seizure when the frequency of the IMF1 begins to drop (*dashed line*). Please note that the pHFOs in the range of 200–500 Hz occur solely when the IMF1 frequency is diminished.

Another example of the pHFOs being present when the IMF1 frequency decreases during seizures is illustrated in Figure 5. The seizure in this animal started with no change in IMF1 frequency presumably because the seizure was volume-conducted to the site of the recording. However, about a third of the way into the seizure, the frequency of the IMF1 decrease and the pHFOs became visible, perhaps indicating that the seizure was no longer propagated to the recording site, but the local neuronal network has become involved in its generation. We found pHFOs in every one of the 17 seizures in which the IMF1 frequencies dropped during the seizures, indicating that the seizures originated at, or close to, the recording site.

### The utility of the power and central frequency of IMF1 for seizure detection

**Figure 6:**
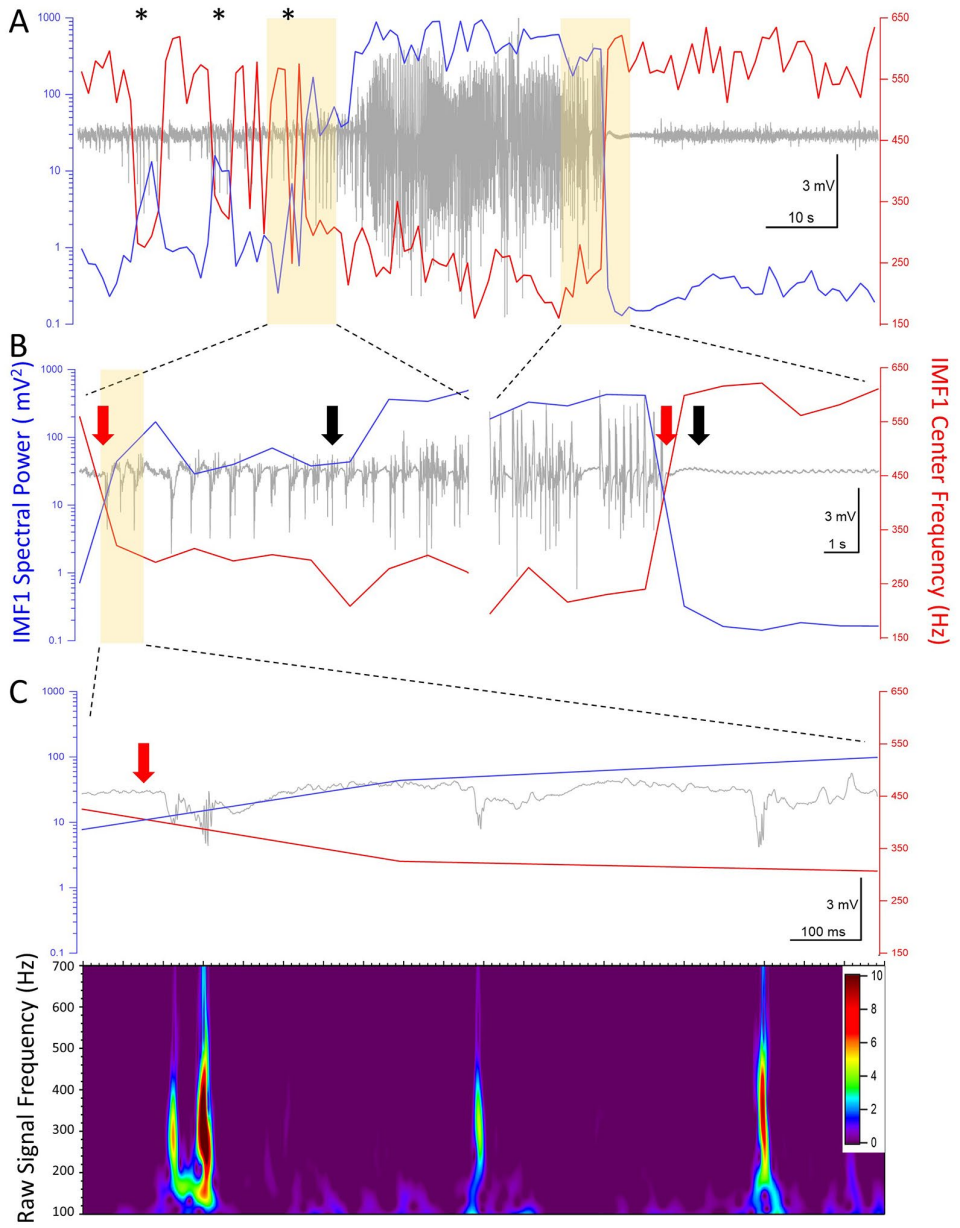
High temporal resolution analysis of the center frequencies and the spectral power (RMS) of IMF1 in the first seizure recorded in animal #I0009. **(A)** Plot of the IMF1 spectral power (*blue line, left y-axis*) and center frequencies (*red line, right y-axis*) at 1 s resolution during the entire pre-seizure, seizure, and post-seizure periods. Note that sudden decreases in center frequencies and commensurate increases in spectral power take place during the pre-ictal period (*asterisks*), but are not sufficiently long-lived to turn into seizures. These phenomena are also manifest at the beginning of the seizure, but last for the entire duration of the seizure, rapidly returning to pre seizure levels immediately after. **(B)** The regions of the recordings highlighted in yellow in **(A)** are shown at a faster time scale. The black arrows indicate the beginning and the end of the seizure as determined by the seizure detection algorithm. The red arrows show the inflection points of the center frequencies and of the spectral power. These precede the black arrows by 6 s at the beginning of the seizure and by 1 s at the end of the seizure, respectively (please see text for details). **(C)** The region of the recording highlighted in yellow in **(B)** are shown at a faster time scale. The three spikes immediately after the center frequency and spectral power inflection points show characteristics of pHFO with frequencies in the 300–500 Hz range. The color scale indicates the Z-score of the Morlet matrix.

Thus far we have shown the aggregate changes in the power and central frequencies during seizures recorded during the epileptogenic period. We next took a closer look at the values of these two parameters on a second-by-second scale, as it has been calculated by our EMD algorithm. Figure 6A shows the raw LFP signal of a seizure in a mouse (I0009), the first electrographic seizure during the epileptogenic period in this animal. In the pre-seizure period, the central frequency of IMF1 shows sudden drops, sometimes for longer than 2 s. During these periods, marked by asterisks above the recordings in Figure 6A, while the frequency of the IMF1 drops, its spectral RMS rises by an order of magnitude. In the raw LFP signal, these periods are characterized by large IIS, but not full electrographic seizures. As shown in more detail in Figure 6B, when the full electrographic seizure begins, the lowered central frequency of the IMF1 does not quickly return to baseline, nor does the massively increased spectral RMS of IMF1. This point, marked by a red arrow in the left panel of Figure 6B, precedes by 6 s the point that marks the beginning of the seizure (black arrow) according to our seizure detection algorithm. Conversely, where our seizure detection algorithm indicated the end of the seizure (black arrow on right panel of Figure 6B), this was 1 s later than the time point where both the IMF1 frequency and spectral RMS returned to pre-seizure values (red arrow on right panel of Figure 6B). In Figure 6C the first three spikes after the decrease in IMF1 frequency and increase in spectral power are shown where on the scale illustrated on the graph, the line of the IMF1 central frequency and of the spectral RMS cross each other (red arrow). The presence of the pHFO (350–450 Hz) characteristic of seizure focus are clearly visible in the Z-scored Morlet wavelet transform of the raw signal between 100–700 Hz (lower panel of Figure 6C).

## Discussion

Our major findings in the present study can be summarized as follows. 1) The EMD analysis of intrahippocampal LFPs recorded at high bandwidth in mice shows exponentially decreasing center frequencies of successive IMFs, much like the named oscillations in the brain (Buzsáki and Draguhn, 2004), 2) During the epileptogenic period induced by suprahippocampal kainic acid administration, electrographic seizures are characterized by decreases in the center frequencies and large increases in the spectral power of the individual IMF components, and 3) The IMF with the highest center frequency (IMF1) drops its frequency during, and even preceding, the seizure. When this drop in frequency happens, there are pHFOs characteristic of the seizure focus, detectable in the pre-ictal and ictal periods.

Compared to the numerous EMD analyses applied to nonstationary EEG recordings (Pachori and Bajaj, 2011; Zahra et al., 2017; Kaleem et al., 2021; Li et al., 2021), ours is the first attempt to derive a single center frequency of the IMFs derived through the analysis. We accomplished this with both a geometrical method that identified an equivalent rectangle to the FFT power spectra of the IMFs (Figure 1C) and by finding the centroid of the FFT. In this manner, we could derive the exponentially declining relationship between these unique center frequency values in successive IMFs (Figure 1D). The meaning of this exponential decline is unclear now, but it may reflect the logarithmic relationship between the frequencies of the various named oscillations found in the brain (Buzsáki and Draguhn, 2004; Buzsáki, 2006).

The EMD analysis has been extensively used in the study of epileptic phenomena in recordings from epileptic patients and from animal models of the disease (Pachori and Bajaj, 2011; Zahra et al., 2017; Kaleem et al., 2021; Li et al., 2021). Usually, the method is used for off-line seizure detection in curated data sets, many of them recorded at a limited bandwidth (Pachori and Bajaj, 2011). Nevertheless, several advances have been made for seizure detection using this method (Cherian and Kanaga, 2022). In most cases of these approaches the first IMF (IMF1) derived by the EMD analysis is considered an artifact and is dismissed as such (Zahra et al., 2017). Because our recordings were of high bandwidth and because of our previous interest in pHFO in this model (Jones et al., 2015), we decided to scrutinize the properties of IMF1. In this process, we discovered that the center frequencies of the IMF1s which are between 400 and 600 Hz in the pre-ictal period decrease by about 35% during the seizures (Figure 2A). Only in one animal in its first three seizures during epileptogenesis did we find that the center frequencies of IMF1 did not decrease during the epectrographic seizures. Most notably, during these recordings whether inter-ictally or during seizures we found no pHFO. The fourth seizure when the center frequency of the IMF1 decreased during the seizure presented both interictal a during seizure pHFO. This finding may have profound consequences for the localization of the seizure focus. Ever since pHFO have been discovered (Bragin et al., 1999), more and more evidence had accumulated that their presence is indicative of a local seizure focus (Bragin et al., 2002; Engel et al., 2009; Ogren et al., 2009; Jefferys et al., 2012). This relationship between pHFO and the focus has been translated into clinical and surgical practice where areas generating pHFO are readily excised during epilepsy surgeries for a better post-surgical outcome for the patients (Weiss et al., 2019). In our study, the presence of pHFO in all recordings where the center frequency of the IMF1 considerably decreased during the seizure compared to the pre-ictal period, may indicate that this behavior of the IMF1 center frequency may also be indicative of a recording from the seizure focus. In this context, the first three seizures in mouse I0001 may have been volume conducted to the recording site, while the fourth seizure may have been genuinely generated close to the recording site. If this can be confirmed in future studies, it may mean that the high frequencies of the IMF1 obtained though EMD analyses of intracranial recordings such as ours, could stem from the highly asynchronous activity of the local neuronal populations surrounding the recording electrode. We may call this a constant desynchronized neuronal “hum,” that gets reduced during the synchronous activity of the cells during seizures. This synchronization would be quite similar to the period doubling Kuramoto model that has been invoked in various brain oscillatory synchronization phenomena (Breakspear et al., 2010) and in numerous synchronization events in biology (Strogatz and Stewart, 1993). The IMF1 frequency reduction may also help the localization of focal seizures and distinguish them from seizures generated elsewhere, but volume-conducted to the site of the recording. It is interesting to note that center frequency reductions coupled to simultaneous spectral RMS increases also occurred pre-ictally (e.g., asterisks in Figure 6A) but these events were somehow prevented from being sufficiently long-lasting to emerge as a seizure. Further analysis will be needed to understand how such events are potentially prevented from turning themselves into seizures.

The timing of the reduction in IMF1 center frequency and the concomitant increase in spectral RMS may also aid in devising novel means for off-line seizure onset and termination criteria. Our modified traditional seizure detector based on (White et al., 2006) performed quite well in detecting the electrographic seizures. However, the changes in center frequency and spectral power of IMF1 would have put the seizure start 6 s and the seizure end 1 s ahead of the respective times defined by the seizure detection algorithm (Figure 6B). It is clear that detection of these critical time points is still ambiguous, and new algorithms based on deep learning may help elucidating this problem (Antonoudiou and Maguire, 2020; Cho and Jang, 2020; Abou Jaoude et al., 2022; Cherian and Kanaga, 2022). In the future, it will be interesting to compare the performance of the deep learning and AI seizure detection approaches to the EMD analyses presented here. If resolving the IMF1 center frequency and spectral RMS could be easily implemented on-line for intracranial recordings and perhaps even for scalp EEG, the findings of our study would be of great use for real time seizure onset detection and ascertaining seizure localization.

## Data availability statement

The raw data supporting the conclusions of this article will be made available by the authors, without undue reservation.

## Ethics statement

The animal study was reviewed and approved by UCLA Chancellor’s Animal Research Committee.

## Author contributions

LM and IM conceived the study. LM analyzed the data, wrote analysis algorithms and procedures, prepared figures, and contributed to the draft of manuscript. IF performed surgeries, obtained recordings, analyzed the data, verified seizure detection, and curated the data. BL analyzed the data and verified seizure detection. PM analyzed and curated the data. JD devised analysis algorithms. IM analyzed the data, wrote analysis algorithms and procedures, prepared final publication quality figures, wrote final version of manuscript, and obtained funding. All authors contributed to the article and approved the submitted version.

## Funding

This work was supported by the following grants and endowment funds to IM. NIH-NINDS, Individual Research Project Grant EUREKA (Exceptional, Unconventional Research Enabling Knowledge Acceleration) 1R01NS75429 “Identifying neurons and circuits critical for epileptogenesis,” and NIH-NINDS, Individual Research Project Grant 1R01 NS030549, “Endogenous GABAergic Activity in the Mammalian Brain,” and the Tony Coelho III Endowment.

## Conflict of interest

The authors declare that the research was conducted in the absence of any commercial or financial relationships that could be construed as a potential conflict of interest.

## Publisher’s note

All claims expressed in this article are solely those of the authors and do not necessarily represent those of their affiliated organizations, or those of the publisher, the editors and the reviewers. Any product that may be evaluated in this article, or claim that may be made by its manufacturer, is not guaranteed or endorsed by the publisher.
